# Paediatric cerebellar glioblastoma - long-term survival following surgery and adjuvant chemoradiotherapy: A case report and literature review

**DOI:** 10.1016/j.bas.2024.102819

**Published:** 2024-04-25

**Authors:** Matthew I. Sanders, Daniel Gatt, Victoria Lee, Stephen B. Wharton, Veejay Bagga

**Affiliations:** a Department of Neurosurgery, Sheffield Children's NHS Foundation Trust, Sheffield, UK; b Department of Neuro-Oncology, Sheffield Children's NHS Foundation Trust, Sheffield, UK; c Department of Histopathology, Sheffield Teaching Hospitals NHS Foundation Trust, Sheffield, UK

**Keywords:** Paediatric cerebellar glioblastoma, Surgery, Chemoradiotherapy, Progression-free survival

## Abstract

**Introduction:**

Paediatric cerebellar glioblastoma is an exceptionally rare clinical entity, with very few cases described in the literature. In the majority of reported cases, prognosis is extremely poor, despite surgical and oncological management. The paucity of data results in lack of consensus as to the optimal management of these patients, with the objective of prolonging survival.

**Research question:**

Do patient or tumour characteristics suggest more favourable rates of progression-free survival in paediatric cerebellar glioblastoma?

**Material and methods:**

Tumour histopathology plus retrospective molecular analysis of archived samples, as well treatment strategy and patient characteristics of a six-year-old child with cerebellar glioblastoma and prolonged progression-free survival were assessed. Characteristics in the published literature that inferred prolonged survival were identified and compared.

**Results:**

Paediatric cerebellar glioblastoma is extremely rare, with only a handful of cases reported over several decades, during which time diagnostic and therapeutic techniques have evolved markedly. Consequently, the scarcity of data with sufficient granularity means that limited conclusions can be drawn. Specific clinical and histopathological factors (i.e. female sex, young age, EGFR negativity and surgical resection plus adjuvant chemoradiotherapy) may indicate a more favourable progression-free survival.

**Discussion and conclusion:**

Rates of progression-free survival in this rare condition are generally poor, however, several patient and tumour characteristics may infer more favourable prognosis. As increasingly refined means of diagnosis and characterisation are developed, particularly as a result of advances in molecular analyses, more adjuvant treatment options are likely to come on stream in future.

## Highlights

• Cerebellar glioblastomas in children are exceptionally rare with no consensus on treatment strategy.
• This review synthesises patient features and treatment strategies in the few cases of prolonged survival.
• Female sex, young age at presentation, EGFR-negativity imply more favourable outcomes.
• Patients should be offered surgery plus adjuvant chemoradiotherapy.

## 1 Introduction

Pilocytic astrocytomas and medulloblastomas are the most common paediatric intracranial tumours (Reddy et al., 2013). Glioblastomas in children are rare and have been reported with an incidence of less than 3% (Dohrmann et al., 1976). At all ages, cerebellar glioblastoma is extremely rare, accounting for 1% of all glioblastoma cases in adults (Hur et al., 2008), whereas in children few cases have been described (Kulkarni et al., 1999; Kalina, 2012; Reddy et al., 2013). Prognosis is often poor despite surgery and adjuvant therapy. We report the case of a six-year-old girl who was diagnosed with cerebellar glioblastoma. Follow-up imaging to date shows no tumour recurrence and she remains clinically stable without evidence of disease progression 10 years following debulking and adjuvant chemoradiotherapy.

In addition, we provide a literature review of paediatric cerebellar glioblastomas and discuss the few cases of long-term survival.

## 2 Case report

**Fig. 1:**
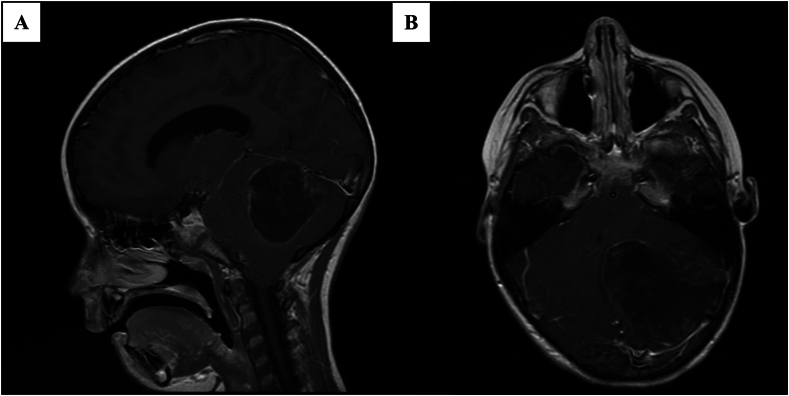
T1-Weighted sagittal (A) and axial (B) MRI images show a large mass occupying majority of the left cerebellar hemisphere. Small focal areas of necrosis are seen within the superior and lateral aspect of the mass. Some contrast enhancement of the margin of the mass is seen, however, the majority of the mass within the centre of the lesion does not show any significant enhancement. There is an inferior descent of the cerebellar tonsils with obliteration of the CSF spaces at the craniocervical junction.

A six-year-old girl presented to her local hospital with a six-week history of progressive lethargy and headache. Neurological examination demonstrated subtle cerebellar signs and papilloedema. A CT scan revealed a posterior fossa tumour with 4th ventricle effacement and obstructive hydrocephalus. She was referred to her local neurosurgical unit where she underwent an emergency endoscopic third ventriculostomy. MRI of the brain (Fig. 1) and spine was performed, which did not demonstrate disseminated disease. She subsequently underwent posterior fossa craniectomy and gross total debulking - a small residuum of tumour was adherent to the dorsal brainstem. She required additional surgery to further treat her hydrocephalus and underwent insertion of a ventriculo-peritioneal shunt.

**Fig. 2:**
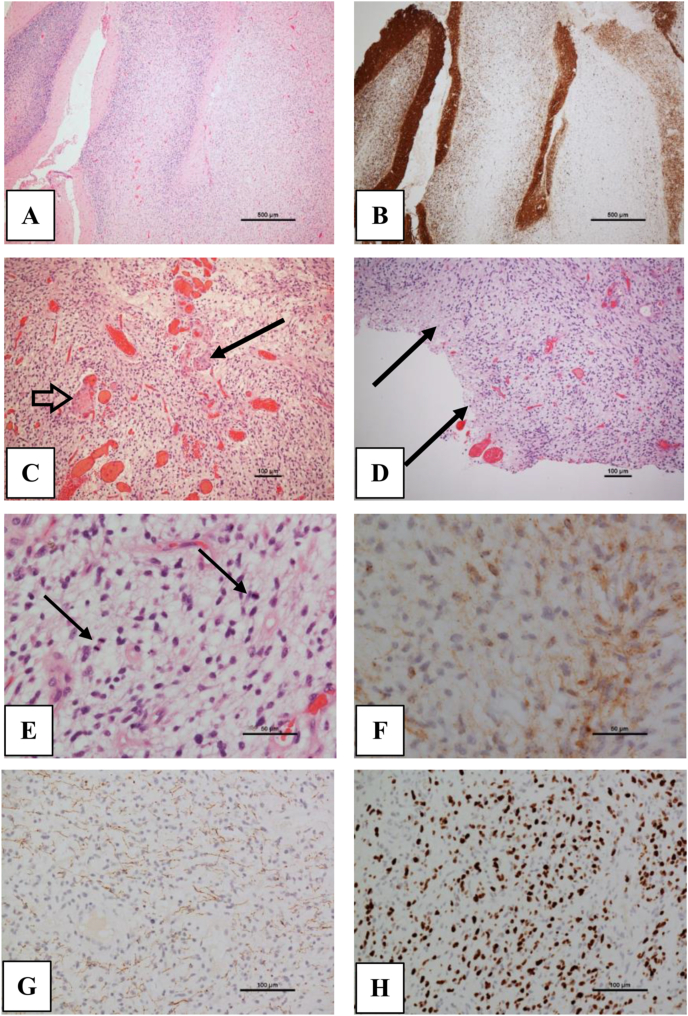
(A) Low power view showing diffuse infiltration into cerebellar folia. The folium on the left is preserved, some residual structures remain in the folium in the middle whilst the architecture of the folium on the right is completed effaced. (B) Immunohistochemistry to synaptophysin highlights the respective preservation, partial loss and effacement of cerebellar structure seen in A. The preserved folium on the left shows a normal pattern with synaptophysin labelling synaptic structures in the molecular (outer) layer and granule cell layer. (C) The tumour has the architecture of a typical diffuse astrocytoma. Note numerous dilated vessels, focally showing true microvascular proliferation (arrow) and thrombosis (open arrow). (D) Necrosis is present at the edge of the tumour fragment. (E) Tumour cells in this area have small pleomorphic nuclei with delicate fibrillary processes in a myxoid background. Two mitoses (arrows) are present in this field. (F) Immunohistochemistry for GFAP (brown signal) labels only a proportion of tumour cells. (G) Immunohistochemistry to neurofilament protein labels residual axons in infiltrated tissue. (H) The tumour shows a very brisk Ki67 labelling index. (For interpretation of the references to colour in this figure legend, the reader is referred to the Web version of this article.)

Histological examination revealed an astroglial tumour of moderate-to-high cellularity with a diffusely infiltrative pattern (Fig. 2). In places the background appeared myxoid. The tumour did not show perivascular condensation, biphasic pattern or rosetting. No small cell (i.e. PNET-like) areas were present. The neoplastic cells showed nuclear hyperchromasia and pleomorphism. Cell processes were present, consistent with the astroglial phenotype, but piloid cells were not seen. Mitotic figures were frequent and there were abnormal vessels with true microvascular proliferation. Small areas of necrosis were identified at the edge of some of the tumour fragments. Immunohistochemical studies demonstrated variable expression of glial fibrillary acidic protein in only a proportion of tumour cells. Neuronal markers labelled only residual elements of cerebellar tissue, highlighting the infiltration. The tumour demonstrated very brisk labelling (>50%) with antibodies to the Ki67 proliferation marker. Immunohistochemistry to IDH1 mutant protein (R132H) was negative. A histological diagnosis of Glioblastoma, WHO grade 4, was made.

Molecular and additional immunohistochemistry assessments were performed retrospectively on the stored tumour. These revealed retention of nuclear ATRX expression. An initial sequencing panel confirmed no mutation in IDH1 or IDH2, with no BRAF, TERT promoter or histone mutations. Low level monosomy 10 was detected. There was no evidence of 1p/19q co-deletion, nor was there evidence of EGFR amplification. There was no evidence of CDKN2A loss or KIAA1549-BRAF gene fusion. MGMT methylation was equivocal. Methylation array was performed using the Infinium Methylation EPIC BeadChip array and data submitted for methylation profiling. This returned no match with the brain tumour classifier (v12.8) but identified multiple copy number variations with: s amplification of MDM4 (1q32.1), PDGRFA (4q12); gains of chromosomes 2, 8 and 19; loss of chromosomes 11 and 13 (including RB1). Further assessment was performed for mutations with the Illumina TSO500 cancer panel. This identified no driver variants are fusions. These results facilitate a final integrated diagnosis of cerebellar glioblastoma, IDH-wild type, WHO grade 4.

**Fig. 3:**
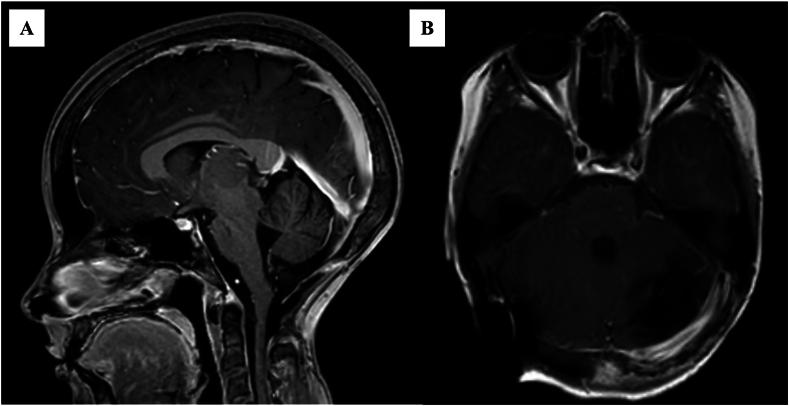
T1-Weighted sagittal (A) and axial (B) images performed in May 2023 (10 years postoperatively), which were reported as showing stable intracranial appearances without evidence of abnormal enhancement.

The patient subsequently commenced a six-week course of radiotherapy at a dose of 54Gy given in 30 fractions to the posterior fossa with temozolomide chemotherapy at 75mg/m^2^. This was followed by a further six courses of temozolomide at 200mg/m^2^ every 28 days. Follow-up imaging was performed at three-monthly intervals for three years, then relaxed to annual and ultimately two-yearly intervals. To date, 10 years postoperatively, follow-up imaging has demonstrated no tumour recurrence. The most recent imaging was performed in May 2023 (Fig. 3).

Assessments at annual outpatient follow-up have been unremarkable, without clinical evidence of disease progression. The patient has also undergone serial neuropsychological assessments. This has included assessment of academic and intellectual ability (Wechsler Intelligence Scale for Children 5th UK Edition), which was within the average range (61st percentile). The patient attends mainstream education. There were deficits in memory, particularly in recall of verbal material. Fine motor skills, executive function and emotional wellbeing were also assessed and fell within average ranges.

## 3 Literature review

Primary cerebellar glioblastoma in children is an extremely rare entity. Accordingly, there are few reported cases where there is adequate or sufficiently disaggregated data suitable for meta-analysis. This results in challenges when drawing conclusions regarding optimal treatment. Our review, therefore, is largely narrative in nature. We do, however, identify trends in demographics and treatment strategies.

In one study, two cases were diagnosed out of a total of nearly 500 intracranial tumours (Dohrmann et al., 1976). A further four cases were reported in a publication which retrospectively assessed a 44-year period (Kulkarni et al., 1999). A more recent case series describes five cases over nearly a decade (Reddy et al., 2013). Although all five of these patients were histologically diagnosed with glioblastoma, one patient had a history of medulloblastoma. Whilst gross total resection (GTR) was reportedly achieved, transformation cannot be excluded and has been described (Schmidbauer et al., 1987). The comprehensive literature review by Reddy et al. (2013) of all paediatric posterior fossa malignant astrocytomas summarises 46 cases (including those previously described by Kulkarni et al.), however, of these, only 16 had a diagnosis of primary cerebellar glioblastoma.

In total, including our report, 60 cases were identified (Table 1). Demographic data were not consistently provided, however, where given, the ratio of female-to-male was similar (1.2:1, with 22 females, 19 males and 19 unspecified). Similarly, specific breakdown of age was not consistently detailed – where published, the mean age at presentation was nine years (range 1.8–17). Despite its rarity, the literature consistently describes the onset and progression of symptoms as rapid. Disease location has been demonstrated to impact survival, with a multivariate analysis by Karremann et al. (2013) comparing cerebellar and supratentorial glioblastoma showing significantly worse survival in the cerebellar group (p = 0.037). The literature also suggests that prognosis is poor despite surgical intervention and adjuvant chemoradiotherapy, with mean survival being approximately 15 months - this is attributed to early recurrence and/or CSF dissemination.

**Table 1:** Summary of literature review of paediatric primary cerebellar glioblastoma.

Reference	Article Summary
Sanders et al., 2024	Case report of a 6-year-old girl with cerebellar glioblastoma who underwent GTR followed by chemoradiotherapy. Patient is alive and clinically well 10 years following her initial presentation.
Dohrmann et al. (1976)	Retrospective study of 488 patients. Two cases of cerebellar glioblastoma identified. Both patients were male with mean age of 5 years. One patient underwent surgical resection followed by radiotherapy while the other patient received no treatment. Survival was 17 & two months, respectively.
Kulkarni et al. (1999)	Retrospective study identifying four patients with cerebellar glioblastoma of which three were male and one female aged 1.8, 15, 1.8 and nine years, respectively. The first patient underwent GTR only and survived 13 months. The second patient underwent STR followed by chemoradiotherapy and survived 15 months. The third patient underwent GTR followed by chemotherapy and was alive at eight-month follow up but had local recurrence. The female patient had GTR with radiotherapy and survived 17 months.
Reddy et al. (2013)	Retrospective study of five paediatric patients with cerebellar glioblastoma, with two males and three females aged 11, 14, 5, 3 & 3 years, respectively. The two male patients had STR followed by radiotherapy. Survival duration was nine & four months respectively. All female patients had GTR. The first female patient was lost to follow-up and information about chemoradiotherapy is not available. The latter two female patients both received post-operative radiotherapy and temozolomide chemotherapy. Survival duration was 3.5 years and 10 months respectively. The article also has an accompanying literature review of 55paediatric patients with posterior fossa malignant astrocytomas, which include some patients with primary cerebellar glioblastomas.
Saito et al. (2006)	Retrospective study analysing the correlation of EGFR expression in glioblastoma and survival outcomes. The paper identified one 8-year-old female patient diagnosed with a cerebellar glioblastoma. She underwent STR and chemoradiotherapy. Patient was still alive at follow up at 64.5 months.
Bristot et al., 1998	Case series of four patients with malignant cerebellar astrocytomas. One patient, a 14-year-old female was histologically diagnosed with a cerebellar glioblastoma. She underwent GTR but died seven days after presentation. *The data maybe misleading as the clinical history, histology and treatment of each patient does not match the data inTable 1*of the article.*
Katz et al. (1995)	Case report of a 4.5-year-old female with a cerebellar glioblastoma. Patient underwent surgical resection. No information on post-operative chemoradiotherapy or survival duration given. The article discusses the MR imaging findings.
Kuroiwa et al. (1995)	Retrospective study analysing the MR findings in nine patients with posterior fossa glioblastoma. Three paediatric patients with cerebellar glioblastoma are included in this study. One female aged two years and two males aged seven & six years, respectively. No data on treatment or survival available.
Mahvash et al. (2011)	Retrospective study analysing paediatric glioblastoma. Two cases of primary cerebellar glioblastoma identified. One 5-year-old female underwent GTR followed by chemoradiotherapy. One 7-year-old male underwent STR followed by chemoradiotherapy. Survival duration was 225 and 21 weeks respectively.
Chamberlain et al., 1990	Retrospective study of 18 patients with poorly differentiated gliomas of the cerebellum. Of these, two paediatric patients with primary cerebellar glioblastoma were identified. The sex of the patients is not detailed. One patient aged nine years underwent GTR. The other patient aged 16 years underwent STR. Both received adjuvant chemoradiotherapy. Survival duration was 14 & 16 months, respectively.
Georges et al. (1983)	Case report of a 17-year-old with a cerebellar glioblastoma who underwent subtotal resection and postoperative radiotherapy. Survival duration was eight months.
Pang and Ashmead, 1982	Case report of a 7-year-old with a cerebellar glioblastoma underwent GTR with post-operative radiotherapy. Recurrence was treated with chemotherapy. The case also describes extraneural metastasis of glioblastoma, which is unusual. Survival was 19 months.
Kopelson et al., 1982	Retrospective study of eight patients diagnosed with cerebellar glioblastoma. One patient was a 10-year-old female who underwent GTR. Survival duration was one month
Kalina, 2012	Case report of an 11-year-old male with a haemorrhagic cerebellar glioblastoma. Patient underwent GTR of the tumour followed by external beam radiotherapy and temozolomide chemotherapy. No details of survival duration were given.
Salazar, 1981	Retrospective study of 13 patients with malignant astrocytomas of the cerebellum including one 13-year-old female patient with a cerebellar glioblastoma who underwent craniospinal radiotherapy. Survival duration was 11 months.
Shinoda et al. (1989)	Case series of five paediatric patients with malignant cerebellar astrocytomas. One male and two females patients aged 13, 10 & 7, respectively, were diagnosed with cerebellar glioblastoma. All underwent surgical intervention with resection rates of 90%, 70% & 70%, respectively. All patients received chemoradiotherapy. Survival duration was seven, 13 & five months, respectively.
Fresh et al. (1976)	Case report of a 9-year-old with a cerebellar glioblastoma who underwent GTR and postoperative radiotherapy. Two further recurrences were treated with further surgery, followed by chemotherapy after the third resection. Survival duration was 26 months. The patient died from gram-negative sepsis secondary to pneumonia.
Luccarelli et al., 1980	Case series of patients diagnosed with cerebellar glioblastoma, of which one patient was a 6-year-old female who underwent STR. Survival duration was three days.
Karremann et al. (2013)	Retrospective study comparing outcomes and predictors of survival between cerebellar and supratentorial cortical HGGs in children. Of the 29 children identified with cerebellar HGG, 17 had a diagnosis of cerebellar glioblastoma. Mean overall survival in cerebellar glioblastoma was significantly worse than cortical glioblastoma, with patients surviving 0.9 ± 0.03 and 1.53 ± 0.23 years, respectively (p = 0.0079).
Papaevangelou et al., 2017	Case report of 7-year-old female with cerebellar glioblastoma and adjuvant chemoradiotherapy. Survival duration was 20 months.

**Fig. 4:**
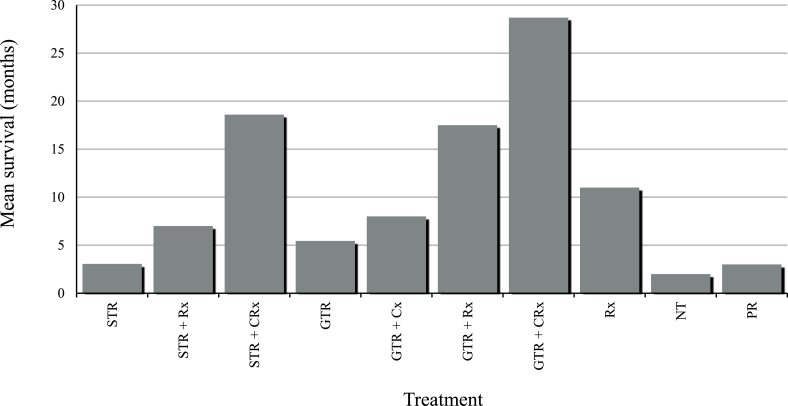
Graph showing mean survival duration following different treatment regimes (where reported). STR, subtotal resection; GTR, gross total resection; CRx, chemoradiotherapy; Rx, radiotherapy; PR, partial resection; NT, no treatment.

Sufficient granularity of data to allow comparison between degree of resection and survival duration was available in 36 cases (Fig. 4). Analysis of the data shows that the patients who underwent GTR plus adjuvant chemoradiotherapy had a longer mean survival compared to patients who underwent subtotal resection (STR) plus adjuvant chemoradiotherapy (28.7 months vs 18.6 months, respectively). This is consistent with work by Mahvash et al. (2011). Whilst survival in patients who underwent GTR alone inferred prolonged survival when compared to STR alone (5.45 months vs 3.05 months, respectively), the number of patients included in these subgroups are small (two and five, respectively) making it difficult to draw meaningful conclusions. Furthermore, one patient who underwent STR alone died due to procedural complications three days post-operatively (Luccarelli, 1980), impacting average survival in that subgroup.

Adjuvant chemoradiotherapy also plays an important role in survival. Mean survival of patients in the GTR-only subgroup was 5.45 months compared to 28.7 months, 17.5 months and 8 months in the GTR + chemoradiotherapy, GTR + radiotherapy and GTR + chemotherapy subgroups, respectively (Fig. 4). The role of adjuvant chemoradiotherapy in prolonging survival following STR has been questioned, as a significant survival benefit was not seen in one study (Mahvash et al., 2011). This does not seem to be consistent with our data as those patients who received adjuvant chemoradiotherapy or radiotherapy alone following STR survived longer than those from the GTR-only group (18.6 months vs 7 months vs 5.45 months, respectively). If there was no benefit from chemoradiotherapy in patients who have undergone STR, then one would expect a worse outcome when compared to patients undergoing GTR-only - this is not borne out by the published data. In fact, one long-term survivor underwent STR with adjuvant chemoradiotherapy, strengthening the case for its treatment role (Table 2).

**Table 2:** Cases of long-term survival (>24 months) in paediatric patients diagnosed with primary cerebellar glioblastoma.

Reference	Age (years)	Sex	Extent of Resection	MGMT Promotor Methylation Status	Chemotherapy Regime	Radiotherapy Regime	Survival (years)
**Sanders et al., 2024**	6	Female	GTR	Equivocal	Temozolomide - six courses at 75mg/m^2^ followed by six courses of temozolomide at 200mg/m^2^ every 28 days	54Gy in 30 fractions to posterior fossa	Alive at 10 years
Reddy et al., 2013	3	Female	GTR	Not stated	Temozolomide – six courses over approx. one-year	59Gy to tumour bed	Alive at 3.5 years
**Mahvash et al. (2011)**	5	Female	GTR	Not stated	Chemotherapy given but agents not stated	Not stated	Alive at 4 years
**Saito et al. (2006)**	8	Female	STR	Not stated	Ifosfamide (900mg/m^2^ on days one-to-five) Cisplatin (20 mg/m^2^ on days one-to-five) and Etoposide (60 mg/m^2^ on days one-to-five)	Whole Brain: 60Gy Local: 14Gy Given in 30 fractions	Alive at 5.4 years
**Fresh et al. (1976)**	9	Female	GTR followed by two further STRs for recurrence	Not stated	CCNU (Lomustine) 200mg every six weeks + Vincristine 0.6mg every two weeks	60Gy to tumour bed	Dead at 2.25 years (due to sepsis)
**Wang et al. (2021)**	11	Female	STR	Negative	Temozolomide – regime not specified	Not individually specified (range of 55.6–56.9Gy locally across series)	Alive at 2.25 years
**Wang et al. (2021)**	10	Female	GTR	Positive	Temozolomide – regime not specified	Not individually specified (range of 55.6–56.9Gy locally across series)	Alive at 4 years
**Wang et al. (2021)**	12	Female	GTR	Negative	Temozolomide – regime not specified	Not individually specified (range of 55.6–56.9Gy locally across series)	Alive at 2.75 years

## 4 Discussion

The rare nature of this condition has resulted in a scarcity of high-quality publications that contain sufficiently granular data. In several publications basic demographic data such as age and sex are not provided. Furthermore, several cases reported were published almost 50 years ago, in which time the management of high-grade gliomas has been revolutionised by diagnostic and therapeutic advances. These factors significantly limit the utility of some publications when trying to draw meaningful conclusions as to what factors might suggest a better prognosis. Whilst interpretation of these results should be taken cautiously, we have, nonetheless, not excluded these cases as they both highlight the rarity of this condition and may still provide useful insight. For example, Dohrmann et al. (1976) reported two cases of paediatric cerebellar glioblastoma, whose management and survival differed. One patient died 17 months following GTR whilst another, who was untreated, died two months following diagnosis. An extensive summary of reported cases can be found in Table 1.

With respect to the case that we report, the astroglial differentiation of the tumour as well as its location, an important histological differential diagnosis is pilocytic astrocytoma, which typically shows cytological pleomorphism and microvascular proliferation. Necrosis can occasionally be seen. The specimen showed none of the architectural features of that entity, however, with no Rosenthal fibres or granular bodies identified. Furthermore, the tumour demonstrated very brisk proliferation in keeping with its high grade. Given the diffuse architecture, lack of any specific features and very high proliferation, histological appearances were not in keeping with those of pilocytic astrocytoma (or the rare entity of malignant progression) and were instead typical of glioblastoma.

Morphology of glioblastoma can now be more accurately defined with molecular analyses, including presence of histone mutations, the diffuse paediatric-type high-grade glioma H3-wildtype, as well as IDH wildtype (Louis et al., 2021). In the case described in this report, molecular analysis did not allow definition of a more specific entity. It is likely that the DNA in the archived sample was of insufficient quality. Hence, using current WHO terminology, the term paediatric-type diffuse high-grade glioma, NOS could be applied.

Another example of variation within glioblastoma tumour genetics is highlighted by the use of temozolomide. Temozolomide is a relatively novel but important chemotherapy agent that has shown to be well tolerated and improve survival when used in conjunction with radiotherapy in both adults (Stupp et al., 2009) and children (Mallick et al., 2015). It functions by causing methylation of DNA, thereby triggering cell death. Glioblastoma have innate repair mechanisms to protect against injury, which is mediated through the O-6-methylguanine-DNA methyltransferase (MGMT) gene. In some glioblastoma, silencing of the MGMT gene by MGMT promoter methylation renders these tumours more sensitive to temozolomide (Weller et al., 2010). If MGMT promoter methylation status of glioblastoma is negative, then these tumours will not respond to temozolomide chemotherapy. The genetic variations observed in the tumours will, therefore, dictate their response to chemoradiotherapy and may also explain the differences reported by Mahvash et al. (2011). Of the 10 cases reported by Wang et al. (2021), only two patients had positive MGMT promotor methylation status and of these two patients, only one was treated with adjuvant temozolomide - this patient was alive at 48 months follow up (Wang et al., 2021). The MGMT promoter methylation status of our reported patient is equivocal, and so it is difficult to comment with any certainty as to what role MGMT status played in the response to temozolomide in this case.

It is difficult to explain why a benefit from adjuvant chemoradiotherapy was not seen following STR in the paper by Mahvash et al. (2011). As the authors of this paper looked at supratentorial and infratentorial glioblastoma (including glioblastoma of brainstem), it may reflect differences in the properties and behaviours of glioblastoma at different locations, and perhaps those located in the cerebellum are more susceptible to chemotherapy and radiotherapy agents. For example, it is recognised that EGFR (epidermal growth factor receptor) expression in glioblastoma varies according to tumour location, and that overexpression of this gene is associated with poorer outcomes as they are less sensitive to radiotherapy. Supratentorial glioblastoma were shown to overexpress EGFR when compared to cerebellar glioblastoma (Saito et al., 2006), which may explain the differences seen in response to adjuvant therapy. We note that the sample in our presented case did not express EGFR, and of the other long-term survivors, two were negative for EGFR expression (Saito et al., 2006; Reddy et al.*, 2013*), whereas two other reported cases do not disclose EGFR immunoreactivity status.

Extent of tumour resection is another important factor. Gross total resection with adjuvant chemoradiotherapy infers prolonged survival when compared to (STR) in paediatric patients with supratentorial or infratentorial glioblastoma (Mahvash et al., 2011). This would appear logical as clinical deterioration is associated with disease recurrence. Conversely, Reddy et al. (2013) reported no significant correlation between the extent of resection and survival in their series. Although conflicting, it is important to highlight that Reddy et al. describe tumours of different grades and not solely glioblastoma, which may explain the differences in reported outcomes.

The prognosis in cerebellar glioblastoma may not be as dismal as previously described (Reddy et al., 2013). The range of survival duration varies from days to years. 13% of patients diagnosed with cerebellar glioblastoma are relatively long-term survivors, having survived over 24 months (Table 2). The prognostic indicators for long-term survival are currently unknown, however, all long-term survivors described are female with a mean age of eight years (range 3–12) at presentation. Young, female patients with primary cerebellar glioblastoma may, therefore, have a more optimistic prognosis. It is important to question, however, whether publication bias has influenced survival data. For what many clinicians might consider a dismal diagnosis, the true mortality rate may be underreported. We hope that this review prompts the publication of unreported cases so a greater understanding of the true natural history of this condition can be achieved. Reporting outcomes of similar cases can be facilitated with the use of registries, both national and multinational, with dedicated discussion at scientific meetings. Collection of standardised, contemporaneous data with sufficient granularity would help identify patterns in presentation, prognostic markers and treatment options.

## 5 Conclusions

We discuss a case of long-term survival in a child diagnosed with primary cerebellar glioblastoma. A literature review is provided and possible prognostic indicators are discussed. As this condition is rare, drawing conclusions from the small sample size is challenging. The behaviour and outcome of primary paediatric cerebellar glioblastoma is highly variable and unpredictable with even poorer rates of survival than supratentorial glioblastoma. Indictors that may favour prolonged survival include female sex of young age, EGFR negativity and GTR with adjuvant chemoradiotherapy. Further analysis of molecular tumour data might elucidate prognostic factors, both in terms of survival and response to adjuvant therapies.

## Funding details

No funding was sought or awarded

## Ethical statement

All identifiable patient data has been anonymised. Written informed consent was obtained from the patient's mother.

## Declaration of competing interest

The authors report there are no competing interests to declare.
